# Pregnancy worsens the morbidity of COVID-19 and this effect becomes more prominent as pregnancy advances

**DOI:** 10.4274/tjod.galenos.2020.38924

**Published:** 2020-10-02

**Authors:** Niyazi Tug, Murat Yassa, Emre Köle, Önder Sakin, Merve Çakır Köle, Ateş Karateke, Nurettin Yiyit, Emre Yavuz, Pınar Birol, Doğuş Budak, Ömer Kol, Edip Emir

**Affiliations:** 1 University of Health Sciences Turkey, Şehit Prof. Dr. İlhan Varank Sancaktepe Training and Research Hospital, Clinic of Obstetrics and Gynecology, İstanbul, Turkey; 2 Darıca Farabi Training and Research Hospital, Clinic of Obstetrics and Gynecology, Kocaeli, Turkey; 3 University of Health Sciences Turkey, Kartal Dr. Lütfi Kırdar Training and Research Hospital, Clinic of Obstetrics and Gynecology, İstanbul, Turkey; 4 İstanbul Medeniyet University, Göztepe Training and Research Hospital, Clinic of Obstetrics and Gynecology, İstanbul, Turkey; 5 University of Health Sciences Turkey, Şehit Prof. Dr. İlhan Varank Sancaktepe Training and Research Hospital, Clinic of Thoracic Surgery, İstanbul, Turkey

**Keywords:** SARS-CoV-2, COVID-19, pregnancy, morbidity, intensive care unit admission, preterm birth

## Abstract

**Objective:**

To investigate pregnancy outcomes and compare the clinical characteristics of coronavirus 2019 (COVID-19) disease in pregnant and agematched non-pregnant women.

**Materials and Methods:**

Hospital records of four tertiary care centers were reviewed retrospectively. The subjects comprised 188 pregnant patients and 799 non-pregnant women who were admitted to these hospitals.

**Results:**

Pregnancy significantly affected the clinical severity of COVID-19 and this effect was more prominent in pregnant women at >20 weeks gestation (p<0.001). Rates of oxygen support (10.1% vs 4.8%; p≤0.001), intensive care unit admission (3.2% vs 0.6%; p=0.009), presence of fever (12.8% vs 4.4%; p<0.001), tachypnea (7.0% vs 2.4%; p=0.003) and tachycardia (16.0% vs 1.9%; p<0.001) were significantly more frequent in pregnant women compared with non-pregnant women. Pregnancy was strongly associated with the need for oxygen support [relative risk (RR), 2.125; 95% confidence interval (CI): 1.25-3.60] and admission to the intensive care unit (RR, 5.1; 95% CI: 1.57-16.53) compared with non-pregnant women. Some 14.4% of the pregnant women had co-morbidities. Sixty of the 188 pregnant women (31.9%) delivered during the Severe Acute Respiratory syndrome coronavirus-2 infection, 11 (18.3%) had vaginal deliveries and 49 (81.7%) were by cesarean section. Of these 60 deliveries, 40 (66.7%) were <37 weeks gestation.

**Conclusion:**

Pregnancy worsens the morbidity of COVID-19 and this effect seems to increase as the pregnancy advances, but not the mortality rate.

**PRECIS:** The clinical severity of COVID-19 pneumonia is worse in pregnant women than in non-pregnant women.

## Introduction

The clinical presentation of Severe Acute Respiratory syndrome coronavirus-2 (SARS-CoV-2) infection, which has been responsible for the current pandemic in 2020, varies from asymptomatic to severe disease and age, sex, presence of co-morbidities, and viral load have been suggested to a play role in the severity of this disease^(1,2,3)^. However, less is known about the clinical picture of pregnant women.

There are a limited number of studies in the literature, some suggesting a similar clinical picture of SARS-CoV-2 infection in pregnancy to that of non-pregnant women, and some showing different results. Regarding altered immunity and response to viral infections in pregnancy, one may suppose a different course of the disease in pregnant women^(1,2,3)^. The outcome of pregnancies infected with SARS-CoV-2 is also still unclear^(4)^. In the current study, it was aimed to investigate pregnancy outcomes and compare the clinical characteristics of coronavirus 2019 (COVID-19) disease in pregnant and age-matched non-pregnant women.

## Materials and Methods

In this study, the subjects were pregnant COVID-19 women who admitted to four tertiary care hospitals with symptoms related to pregnancy or COVID-19 and those of non-pregnant women in the same age group (18-45 years) admitted with reasons related to COVID-19 to the same hospitals between March 11^th^ and June 30^th^, 2020. All patients were managed according to the national treatment protocols.

A total of 987 patients who were diagnosed as having COVID-19 were enrolled in the study. Of these, 188 patients were pregnant (19%) and 799 were non-pregnant (81%). Four tertiary centers (Şehit Prof. Dr. İlhan Varank Training and Research Hospital, İstanbul; Kartal Dr. Lütfi Kırdar Training and Research Hospital, İstanbul; Darıca Farabi Training and Research Hospital, Kocaeli; Medeniyet University Hospital, İstanbul) provided data of 557 (105 pregnant), 280 (30 pregnant), 147 (50 pregnant) and three (three pregnant) women diagnosed as having SARS-CoV-2 infection, respectively. There were no excluded patients.

Data were retrospectively collected from health records using a standardized data collection form. Pregnant women diagnosed as having COVID-19 infection up until June 30^th^, 2020, were included as the study group. Data for the clinical, obstetric and laboratory outcomes were regularly updated until July 7^th^, 2020. Primary outcome measures were the comparison of the clinical severity of the COVID-19 infection, mortality, rates of need for intensive care unit (ICU), oxygen support, and clinical and laboratory findings. The secondary outcome measure was the obstetric outcomes. Each institution obtained approval from the local administration board, regional ethical committee, and national scientific research platform (approval no: 20/207, date: 05.06.2020).

The diagnosis of COVID-19 infection was made by either with reverse transcription real time polymerase chain reaction (RT-PCR) testing for SARS-CoV-2 or imaging studies including chest computed tomography, lung ultrasound or chest X-ray. The clinical severity of COVID-19 disease was classified according to the World Health Organization (WHO)^(1)^. Patients with mild-to-moderate disease and severe-to-critical disease were classified as symptomatic patients who did not require oxygen support and those who received oxygen support, respectively. The decision for oxygen support, tracheal intubation and admission to the ICU were made with the consultation with anesthesiology team on clinical grounds. First trimester of pregnancy was defined as <14 weeks, second trimester between 14weeksand 27 weeks and 6 days, and third trimester as ≥28 weeks until birth. Extremely preterm (<28 weeks), very preterm (28-32 completed weeks of gestation), moderate-to-late preterm (>32 - <37), early term (37 - <39), and term (≥39 weeks of gestation) birth was defined according to the WHO classification^(5)^.

### Statistical Analysis

The collected data were analyzed using the SPSS software version 22.0 (IBM Corp., Armonk, NY, USA). The normality of the demographic data was assessed using the Shapiro-Wilk test. Demographic data are summarized as the median ± inter-quartile range for non-normally distributed data and as the mean ± standard deviation for normally distributed data. Pearson’s chi-square test was used to assess whether pregnancy and the clinical severity of the COVID-19 infection were related. Student’s t-test and the chi-square test were used to compare the means and rates of independent groups as indicated. A p-value of <0.05 was considered to indicate a significant difference.

## Results

### Demographic Data

The median age of all patients was 31±12 (range, 18-45) years. In all women, the diagnosis of infection was performed using RT-PCR testing for SARS-CoV-2 in 89.7% (n=885) and the remainder (n=102, 10.3%) was diagnosed only through imaging studies. RT-PCR testing confirmed the infection in 95.7% of pregnant women (n=180) and the diagnosis was confirmed only with imaging studies in 4.3% of pregnant women (n=8).

### Disease Severity

Pregnancy significantly affected disease severity (p≤0.001). Pregnancy was strongly associated with the need for oxygen support [relative risk (RR), 2.125; 95% confidence interval (CI): 1.25-3.60] and admission to the ICU (RR: 5.1; 95% CI: 1.57-16.53) compared with non-pregnant women. Pregnant and non-pregnant patients with mild-to-moderate symptoms and those who needed are O_2_ illustrated in Table 1. A triple comparison is given in Table 2, where the pregnant women were further divided into two according to gestational age (≤ or >20 weeks gestation), and first, second and third-trimester groups are given in Table 3. These data show that besides pregnancy state, gestational age also affects the disease severity.

Among 799 non-pregnant patients, 353 (44.2%) had a positive contact history with a confirmed person with COVID-19 and 446 (55.8%) did not, whereas these values were 57 (30.3%) and 131 (69.7%) in pregnant women, respectively (chi-square test, p=0.001).

### Clinical and Laboratory Features

As given in Table 4, the presence of fever, tachypnea, tachycardia, and admission to ICU rates differed significantly between pregnant women and non-pregnant women. Three non-pregnant women and none of the pregnant women died of COVID-19 (p>0.05). The difference did not reach to the level of significance in the intubation/mechanical ventilation rates in the non-pregnant women and the pregnant women (0.6% and 2.1%, respectively; Fisher’s Exact test, p=0.073). Rates of having normal leucocyte and lymphocyte counts were also worse in pregnant women compared with non-pregnant women (p<0.001, Table 4). None of the patients received extracorporeal membrane oxygenation. Hospitalization period, and serum values of lactate dehydrogenase and D-dimer were found as similar between pregnant and non-pregnant women (Table 5).

### Pregnancy Outcomes

The median gestational week of the pregnant women was 26±20 (range, 5-41) weeks at the time of admission. The median parity of the pregnant women was 1±2 (range, 0-7) pregnancies.

Sixty pregnant women (31.9%) delivered at a median gestation week of 38 (±2 weeks). Of these 60 deliveries, one was extremely preterm (1.7%; 27^th ^week), two were very preterm (3.3%; 31^st^ and 31^st ^weeks), 11 were moderate/late preterm (18.3%), 26 were early term (43.3%), and 20 (33.3%) were term. Eleven (18.3%) of the deliveries were vaginal and 49 (81.7%) were by cesarean section. None of the cesarean operations was performed under general anesthesia. Indications for cesarean delivery were maternal compromise in eight (16.3%), fetal compromise in 21 (42.9%), obstetric reasons (e.g. previous cesarean, failed progress labour) in 18 (36.7%), and maternal request in two (4.1%). Three of the pregnant women had abortion (1.6%) at their 7^th^ gestational week.

No maternal-fetal transmission of SARS-CoV-2 was observed. None of the RT-PCR tests received from all neonates delivered from mothers with COVID-19 (first immediately after birth and second 48 hours later) were positive. None of these neonates manifested any signs related to COVID-19.

Of all 188 pregnant women, 27 (14.4%) had maternal co-morbidities including obesity (n=8), asthma (n=8), chronic bronchitis (n=1), advanced maternal age (n=2), type 1 diabetes mellitus (n=2), chronic hypertension (n=2), hypothyroidism (n=3), hyperthyroidism (n=1), and arrhythmia (n=1). The clinical severity of COVID-19 was not found to differ by means of having any of these maternal co-morbidities in pregnancy (chi-square test =0.809, p=0.667).

Pregnancy was complicated with preeclampsia in six (3.2%) patients, all of whom delivered by cesarean section. For women with COVID-19, two were asymptomatic, one had mild symptoms, one had moderate symptoms but did not receive O_2_, and two were admitted to the ICU. Another two patients (1.1%) had twin pregnancies and received O_2_ support and recovered thereafter. They had ongoing healthy pregnancies at their 14^th^ and 32^nd^ gestational weeks. Guillain-Barré syndrome developed in one patient with moderate disease severity who did not require O_2_ support. One asymptomatic patient had an ectopic pregnancy and was managed surgically because of unstable hemodynamics. Gestational diabetes was diagnosed in one asymptomatic patient who currently had an otherwise normal pregnancy at her 25^th^ gestational week. Placenta previa was diagnosed in two patients (one asymptomatic and one with mild symptoms) who underwent cesarean section.

## Discussion

Debate still exists as to whether pregnancy is adversely affected by the clinical features of COVID-19. Previous studies suggested increased morbidity of the disease in pregnancy, but opposing findings have also been reported^(1,2,3,4)^; in our opinion, all of which had limitations, which makes the results impossible to generalize.

An early study conducted by Barbero et al.^(6) ^on 91 women with SARS-CoV-2 infection during pregnancy and puerperium. Their analysis showed that 40 patients developed pneumonia, bilateral in most cases, with a 46.2% rate of hospitalization and four patients requiring ICU admission. In the UKOSS study, data were made available for 427 pregnant women admitted to hospitals in the United Kingdom with confirmed SARS-CoV-2 infection between March 1^st^ and April 14^th^, 2020. Indications of hospitalization of the subjects were both symptoms of COVID-19 and other obstetric indications with co-existent SARS CoV-2 infection; 38 of 427 women (9%) required level-3 critical care, four women (<1%) received extracorporeal membrane oxygenation, and five women died (1.2%). Forty-two percent were treated successively and discharged whilst still pregnant. On the other hand, 59% of the deliveries consisted of cesarean births (20% received general anaesthesia), nearly half were due to maternal or fetal compromise. They did not make a comparison with non-pregnant women^(7)^. Kasraeian et al.^(4)^ conducted a meta-analysis including 87 pregnant women who were SARS-CoV-2 positive. Mild or moderate symptoms for SARS-CoV-2 were reported as 78%. No vertical transmission was seen and the pregnancy complications did not differ from those of non-infected pregnant women. In their report, no direct comparison was included, but they still put forward a similar pattern of the clinical characteristics of COVID-19 pneumonia in pregnant women to that of other adult populations. They did not stratify the pregnant patients according to gestational age. Breslin et al.^(8)^ reported the results of 43 test-confirmed COVID-19 cases at a pair of affiliated New York city hospitals. The patients were collected either by hospital admission due to the COVID-19 disease symptoms or by universal testing for all obstetrical admissions. Of these, 29 were symptomatic at presentation and three developed severe disease. The authors compared their findings with a previously published study by Wu and McGoogan^(9)^ and concluded that these rates were similar to the rates of the non-pregnant women. Blitz et al.^(10)^ shared data from seven hospitals in New York State between March 2^nd^ and April 9^th^, 2020. After excluding patients with incomplete data, the incidence of ICU admission was compared between pregnant and non-pregnant women with COVID-19 aged between 15-49 years. Of these, 332 were non-pregnant females, and 82 were pregnant females. In all, 50 non-pregnant females (15.1%) and eight pregnant females (9.8%) were admitted to the ICU for worsening respiratory status, with no statistically significant difference.

On the other hand, the Public Health Agency of Sweden analyzed SARS-CoV-2 infection treated in ICUs in Sweden between March 19^th^ and April 20^th^, 2020, and compared pregnant and non-pregnant women of similar age. Fifty-three women aged 20-45 years with SARS-CoV-2 were reported and 13 of these women were either pregnant or postpartum (<1 week). The results indicated that the risk of being admitted to the ICU was greater in pregnant and postpartum women with laboratory-confirmed SARS-CoV-2 compared with non-pregnant women of similar age^(11)^. A similar result was seen in a study conducted by Ellington et al.^(12)^ that analyzed national data of the United States of America. Data on pregnancy states were available for 91,412 women with laboratory-confirmed SARS-CoV-2 infections; among these, 8207 were pregnant. Some 31.5% of pregnant women and 5.8% of non-pregnant women of reproductive age were hospitalized. The risk for ICU admission and intubation were also greater in pregnant women, but the risk for death was similar.

The results of the present study also show pregnancy as a risk factor for COVID-19. A comparison of the confirmed SARS-CoV-2 infected women revealed that pregnant women had significantly higher rates of COVID-19 related symptoms, fever, tachypnea, tachycardia, ICU admission, and abnormalities in leucocyte and lymphocyte counts. Five (0.6%) non-pregnant women and four (2.1%) pregnant women required mechanical ventilation (p>0.05). Three (4%) non-pregnant women and none of the pregnant women died of COVID-19 (p>0.05, Tables 1, 4).

On the other hand, when the pregnancies were further dichotomized according to gestational age, those with >20 weeks gestation represented even worse rates (being asymptomatic, need for O_2_ support, and ICU admission) than those with ≤20 weeks gestation (Tables 2, 3, 6). A significant difference was also found between symptomatic and asymptomatic pregnant women in each trimester of pregnancy (Table 3).

The discrepancy of many studies, including ours with the above-mentioned studies that show no difference in the morbidity of COVID-19 in pregnant women compared with non-pregnant patients in the reproductive age group, could be secondary to the different gestational ages of the included pregnant patients. During pregnancy, besides the alteration in immunity, some anatomic and physiologic changes might make women more vulnerable to SARS-CoV-2 infection. Hyperemia, edema, and friability of mucosa and hypersecretion in the upper respiratory system^(13,14)^ may provide a better route of entry for viruses. Also, the enlarged uterus displaces the diaphragm upwards leaving less room for the lungs to expand, hence leading to decreased functional capacity and residual capacity. Moreover, increased metabolic demands and oxygen^(13,14)^ may decrease the threshold of pregnant women to compensate for the signs and symptoms of SARS-CoV-2 infection.

In a meta-analysis conducted by Khalil et al.^(15)^, 20 studies on pregnant women with RT-PCR-confirmed SARS-CoV-2 involving 2567 women were analyzed. Among all the pregnant women in these studies, 73.9% were in their third trimester; 52.4% had delivered, 48.3% by cesarean section. The rate of preterm birth (<37 weeks) was found as 21.8%, which were mostly (18.4%) iatrogenic. The ICU admission rate was 7.0% and the intubation rate was 3.4%. Maternal mortality was about 1%. Maternal age over 35 years was a risk factor for all co-morbidities and ICU admission. Neonatal nasopharyngeal RT-PCR swabs were positive in 1.4%.

In the present study, 60 of the 188 pregnant women (31.9%) delivered at a median of 38 (±2 weeks) of gestation. Of these 60 deliveries, 40 (67%) were preterm (<37 weeks). Eleven (18.3%) of the deliveries were vaginal and 49 (81.7%) were by cesarean section. None of the cesarean operations was performed under general anesthesia. The indications of the cesareans were maternal compromise in 8 (16.3%), fetal compromise in 21 (42.9%), obstetric reasons (e.g. previous cesarean, failed progress labour) in 18 (36.7%), and maternal request in two (4.1%). Three of the pregnant women had abortion (1.6%) at their ~7^th^ gestational week. No maternal-fetal transmission of SARS-CoV-2 was observed.

In our opinion, the relatively higher rate of preterm delivery seen in our results might be related to the severity of the SARS-CoV-2 infection. Different modes of medications and morbidities in the cohorts of the patients might be the underlying reason for the discrepancies observed in studies involving pregnant women infected with SARS-CoV-2 in the literature. Larger studies are needed to define whether a better controlled maternal disease could decrease the risk of preterm birth in COVID-19.

Currently, all studies in the literature, including ours, have been performed on a hospital basis. In our view, studies in which subjects collected by screening the general population could give the exact rates of the COVID-19 morbidity and mortality.

## Conclusion

Pregnancy worsens the morbidity of COVID-19 and this effect seems to increase as the pregnancy advances, but not the mortality rate.

## Figures and Tables

**Table 1:**
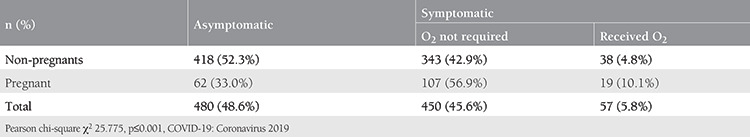
Clinical severity of pregnant and non-pregnant women infected with COVID-19

**Table 2:**
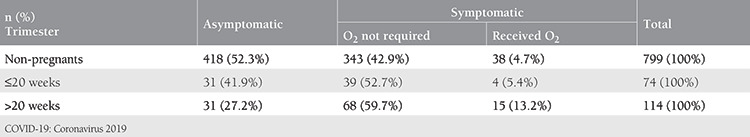
The clinical severity of COVID-19 in non-pregnants and pregnants ≤ or >20 weeks gestation (chi-square test, χ^2^=32.913, p<0.001)

**Table 3:**
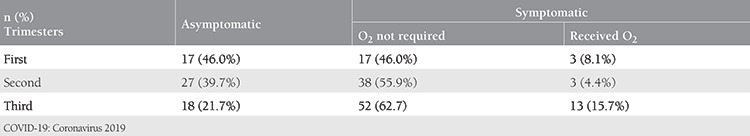
The clinical severity of COVID-19 in the 1^st^, 2^nd^ and 3^rd^ trimesters of pregnancy (Pearson chi-square χ^2^=12.167, p=0.016)

**Table 4:**
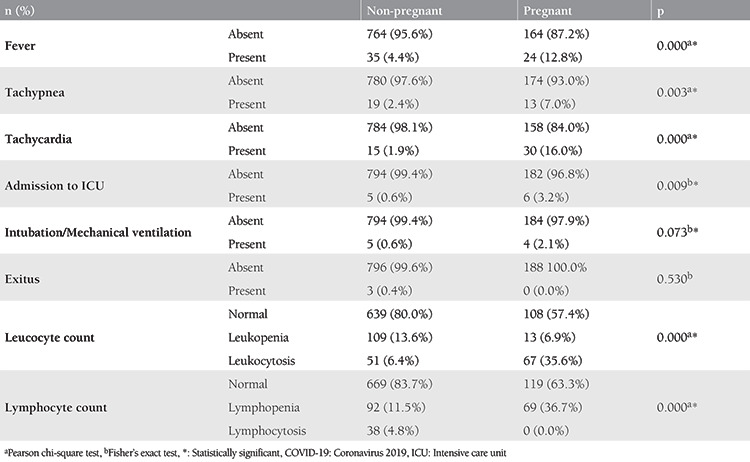
Clinical features and leukocyte and lymphocyte states of the pregnant and non-pregnant women with COVID-19

**Table 5:**
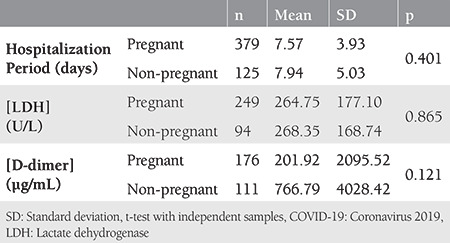
Comparison of symptomatic pregnant and symptomatic non-pregnant women with COVID-19

**Table 6:**
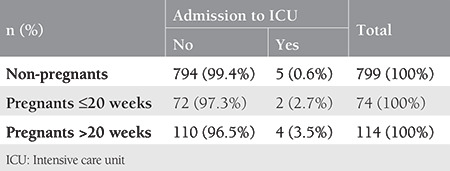
Intensive care unit admission in pregnants ≤ and >20 weeks and non-pregnants (chi-square test, X^2^=9.355, p=0.009)
